# Microbiomes, plausible players or not in alteration of host behavior

**DOI:** 10.3389/fmicb.2014.00775

**Published:** 2015-01-12

**Authors:** David G. Biron, Ludovic Bonhomme, Marianne Coulon, Øyvind Øverli

**Affiliations:** ^1^Laboratoire “Microorganismes: Génome et Environnement,” Clermont Université, Université Blaise PascalClermont-Ferrand, France; ^2^CNRS, UMR 6023, LMGEAubière, France; ^3^INRA, UMR 1095, Genetics, Diversity, and Ecophysiology of CerealsClermont-Ferrand, France; ^4^Department of Biology, UMR Genetics, Diversity and Ecophysiology of Cereals, Université Blaise PascalAubière, France; ^5^Department of Animal and Aquacultural Sciences, Norwegian University of Life SciencesAas, Norway

**Keywords:** alteration of host behavior, manipulative parasite, microbiome, micro-organisms, host-parasite cross-talk

## Introduction

Many parasites can affect the physiology and behavior of their hosts in ways that seem to improve the parasites' chances of completing their life cycle (Biron and Loxdale, 2013; Lafferty and Shaw, 2013; Webster et al., 2013). These parasite species are so-called “manipulative parasites.” Common habitats of manipulative parasites are the host's body cavity, muscles and brain (Lafferty and Shaw, 2013). Typically the host's neural, endocrine, neuromodulatory, and immunomodulatory systems are targeted (Adamo, 2013; Lafferty and Shaw, 2013). In evolutionary biology, manipulation of host behavior by parasites is considered to be an example of the “extended phenotype” concept (Dawkins, 1982; Libersat et al., 2009). There are numerous fascinating cases of alteration of host behavior induced by a parasite; for instance, the suicidal behavior of crickets induced by hairworms (Thomas et al., 2002; Biron and Loxdale, 2013).

Many studies on strategies used by manipulative parasites assume that only two organisms are involved in crosstalk based on Dawkins' assumption: the host and a manipulative parasite. However, hosts are frequently invaded by more than one species of parasite (Ferrari and Vavre, 2011; Cézilly et al., 2014). The interests of different parasitic species may conflict; for example, two parasites may share an intermediate host but require a different definitive host. Parasite-parasite interactions in the intermediate host can result in perturbation of the parasite infection process for each parasite species (Lafferty and Shaw, 2013; Cézilly et al., 2014).

As far as we know, microbiomes are not considered to be taking part in crosstalk between an aquatic host and a manipulative parasite. Therefore, we first briefly present the background for microbiomes as plausible and underestimated players in the crosstalk in host-manipulative parasite associations in aquatic ecosystems, and secondly we discuss concepts and -omics methods to determine whether or not host microbiomes can influence host behavior in aquatic models. Finally, we discuss the importance of considering context-dependent changes in the analysis of -omics data to decode and understand the role of a host microbiome in the alteration of host behavior in aquatic ecosystems.

## Background: microbiomes and animal behavior

Hosts contain distinct habitats where microorganisms and metazoan species like cestodes, nematodes, hairworms, trematodes, and acanthocephalan worms live and compete for resources. The ecological communities of commensal and symbiotic microorganisms (i.e., bacteria, yeasts, fungi, and viruses) living in the internal (example: gut, lachrymo-nasal, respiratory, and urogenital tracts) and epidermal (example: skin, fishes' gills) body surfaces of metazoans are typically considered “normal” or “healthy” microbiomes (Simpson et al., 2005; Mueller et al., 2012; Relman, 2012; Llewellyn et al., 2014). Pioneer studies on microbiomes were done on animal models (example: cow, honeybee, chicken, drosophila, mosquito, mouse, pig, teleost species, zebrafish) and humans (i) to identify microbiomes in healthy individuals; (ii) to decipher microbiome responses to host pathology, parasite invasion, host nutrition and host stress; and (iii) to determine plausible impacts of microbiomes on animal behavior (Smith et al., 2007; Ezenwa et al., 2012; Fagundes et al., 2012; Louis and Flint, 2013; De Palma et al., 2014; Llewellyn et al., 2014; Sison-Mangus et al., 2014; Stilling et al., 2014).

Most microbiome studies have focused on the gut microbiome, because this is a key host habitat for dynamic interactions between the animal host and components of its environment, including nutrients, liquids, and parasites. To date, these studies have revealed that the gut microbiome is involved in key host functions that assist the host in completing its life cycle: for example, (i) prevent parasite invasion of host tissues (example: helminthes, apicomplexa (malaria, sleeping sickness), Vibrio, *Pseudomonas, Streptotoccus*), (ii) nutrition (i.e., aid host digestion by producing molecules helping in food assimilation), and immunomodulation (i.e., stimulation of host immune system favoring an efficient immunity against invasive organisms) (Ringø et al., 1997; Gomez and Balcazar, 2008; Ley et al., 2008; Louis and Flint, 2013; De Palma et al., 2014; Llewellyn et al., 2014; Stilling et al., 2014). Recent research suggests that the host gut microbiome is closely involved in the maturation and functioning of the central nervous system (CNS) of model species (i.e., human being and mouse) by producing and releasing neuroactive molecules (Cryan and Dinan, 2012). Moreover, ethologists observed that behaviors (example: mating, feeding, and anxiety) of many animal species including human beings could be altered by the host gut microbiome (Archie and Theis, 2011; Ezenwa et al., 2012; Lizé et al., 2013; Alcock et al., 2014).

## Manipulative parasites and microbiomes

**Figure 1:**
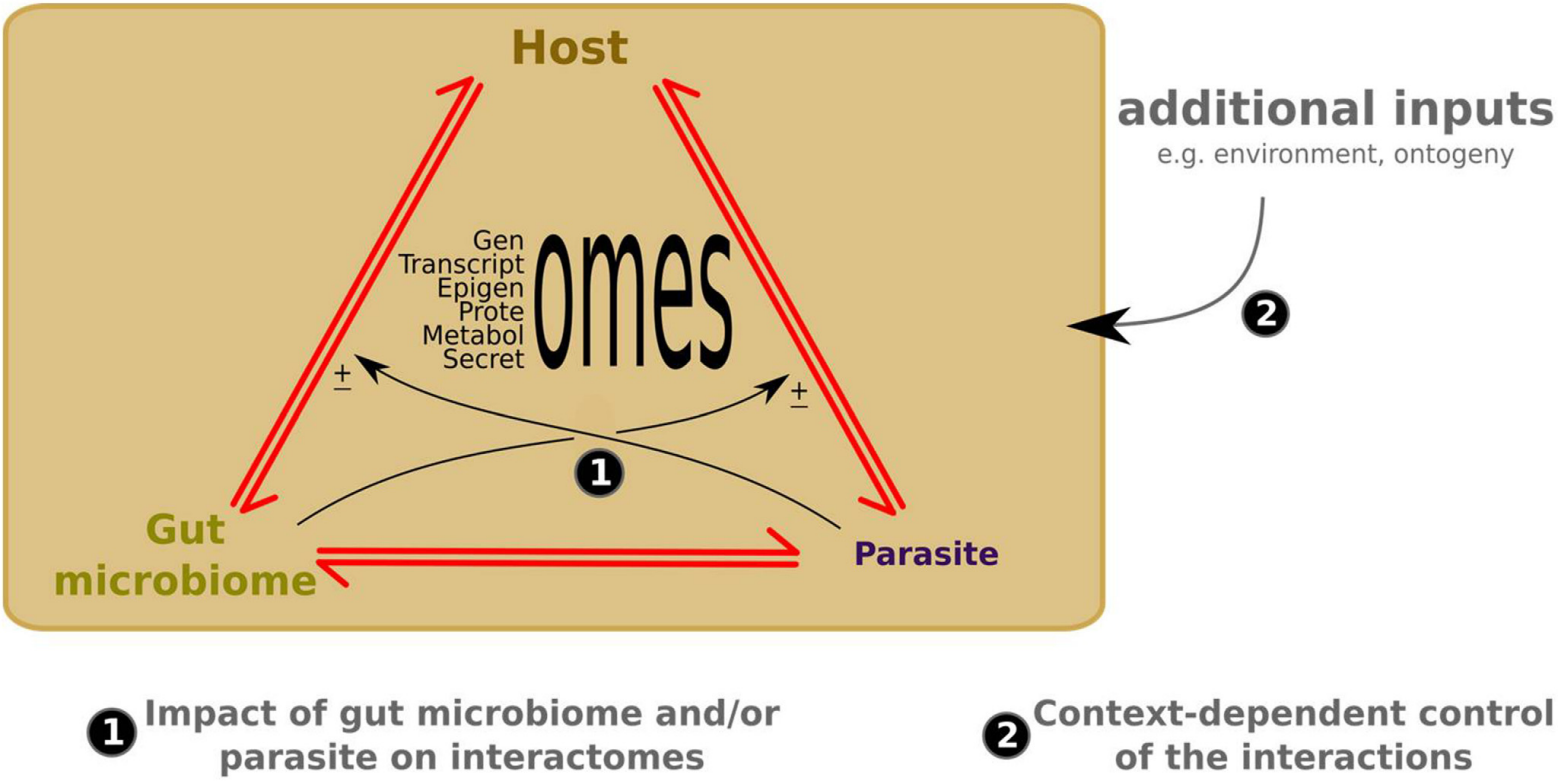
**New -omics and meta-omics technologies help to identify candidate molecular interactions in the three-partner crosstalk (1) and context-dependent control of the interactions (2)**. Red arrows indicate molecular crosstalk between interacting partners; ± refers to possible additive, antagonist or neutral effects of the gut microbiome or parasite on the host/parasite or host/gut microbiome crosstalk, respectively.

Microbiome research is new and mainly focuses on mice and human models (Fagundes et al., 2012; Alcock et al., 2014). However, recent work shows that microbiomes of teleost fish serve as defense against parasitic microorganisms, for example, by preventing the colonization of pathogenic bacteria (example: *Streptococcus* species) via competitive exclusion or via toxic secondary metabolites (Llewellyn et al., 2014). To date, no study suggests that microbiomes can alter the behavior of aquatic hosts but it was demonstrated that a part of the microbiome (i.e., bacterial biofilms) of many marine invertebrates, from corals to sea urchins, play a key role in the settlement behavior of larval stages (Ezenwa et al., 2012; Huang et al., 2012). The understanding of molecular tactics used by microorganisms to modulate the behavior of their hosts results mainly from studies of host-manipulative parasite associations. Many animals use behavioral strategies to avoid manipulative parasite species. Manipulative parasites manipulate host behavior by secreting molecules that act directly and/or indirectly on the maturation and functioning of host CNS (Biron and Loxdale, 2013; Hughes, 2013). However, given the diversity of non-pathogen and beneficial microorganisms in aquatic ecosystems, it is important to expand the view of host behavior/microorganism interactions to include at least the gut microbiome as a third plausible player when a manipulative parasite interacts with its host because the gut microbiome produces neuroactive molecules that can pass via the enteric nervous system (ENS) to interact with the host brain and host nervous system (Gershon, 2008; Fagundes et al., 2012; Schoofs et al., 2014). Thus, cross-talk could be engaged between at least three groups of organisms: the host and its gut microbiome, and an invading manipulative parasite species (Figure 1).

When manipulative parasites are not living in the host's CNS, the mechanisms mediating host behavioral changes are more difficult to determine. Secretion of effective amounts of neurotransmitters by parasites is not easy to confirm (Adamo, 2013). Gammarids may be one example (Maynard et al., 1996; Helluy, 2013). This crustacean family is an intermediate host of many manipulative parasite species (example: trematodes and acanthocephalan worms) (Ponton et al., 2006; Lefèvre et al., 2009; Biron and Loxdale, 2013). The molecular mechanisms used by these parasites (i.e., trematodes and cestodes) to manipulate the host biochemical pathways to alter the host's serotonergic system are still unknown.

Because gut microbiomes can produce neuroactive molecules, we assume that when a manipulative parasite, for instance *Polymorphus paradoxus*, (Acanthocephala: Polymorphidae) invades the gut of *Gammarus lacustris* (Amphipoda, Gammardiae), a part of the gut microbiome would respond to the invasion. Assuming that it is likely that microbiome responses to parasites are conserved between aquatic and terrestrial species, microbiome immune molecules and host immune strategies will target the parasite. During this host/microbiome/manipulative parasite crosstalk, neuroactive molecules produced by the gut microbiome should have additive and/or negative effects during the manipulative process by *P. paradoxus*. Such an effect could occur via a disturbance of the host ENS and/or via the microbiome neuroactive molecules released into the host's hoemcoel. The possible additive effect of the gut microbiome could help answer the key questions regarding how parasites manipulate their hosts and how small metazoan parasites produce enough neuroactive molecules to alter directly or indirectly the host CNS functioning? Whether or not the gut microbiome is involved in these interactions is a fascinating question. This new and promising research avenue will contribute to our general knowledge of molecular crosstalk in host/gut microbiome/parasite relationships and may assist in the search for new methods to treat parasitic diseases.

In order to study host-manipulative parasite associations, there are key experimental steps needed in order to decipher the possible host/gut microbiome/manipulative parasite cross-talk (Figure 1): (i) sampling of host CNS, host ENS, host GM and of the manipulative parasite from laboratory strains and/or from field sampling collection for infected and uninfected hosts (i.e., control) before, during and after manipulation by the parasite; (ii) use of complementary -omics tools (example transcriptomics, proteomics and metabolomics) to reveal the host/gut microbiome/manipulative parasite cross-talk before, during and after the manipulation by the parasite; (iii) analysis of -omics results with specialized software including genome/environment statistical methods to find candidate molecules; (iv) functional analysis (microinjection, immunochemistry, RNAi) and interactome bioassays to confirm or determine the key roles (or not) of the candidate molecules from the three organisms in interactions, and to establish a kinetic map of the biochemical networks of molecules involved in the host/gut microbiome/manipulative parasite cross-talk by using software like cytoscape (http://www.cytoscape.org/). These “-omics” guidelines could help to suggest when the gut microbiome could have additive, antagonist or neutral effects during the manipulation process of a host by a manipulative parasite.

Biological entities named interactomes correspond to the complete set of protein–protein interactions existing between all of the proteins of an organism (Biron et al., 2006). The identification of protein interactions and protein complexes is being increasingly refined in many single and multicellular organisms (Bouveret and Brun, 2012; Braun and Gingras, 2012). However, little is known about large-scale protein interactions between hosts and parasites, and nothing is known about the possible host/gut microbiome/manipulative interactome, although the drawing up of such maps will provide an essential foundation to determine the success or not of molecular strategies used by manipulative parasites to take control of many host cellular functions, and to alter the behavior of their host, which should favor and ensure the continuation of their life cycle.

## Impact of context-dependent changes

Microbiome interactions may be context-dependent. For example, if hosts have resistant or susceptible genotypes and parasites have virulent or avirulent genotypes, are these fixed phenotypes independent of the gut microbiome or, more broadly, independent of the environment? An increasing number of studies suggest that the outcome of host/parasite interactions is not fixed by genetic factors. These studies address the role of exogenous or endogenous factors on the expression of both host and parasite genes during infection (Ferguson and Read, 2002; Thomas and Blanford, 2003; Barrett and Agrawal, 2004; Mitchell et al., 2005; Lambrechts et al., 2006; Salvaudon et al., 2007; Wolinska and King, 2009). If the gut microbiome is involved in host/parasite interactions including the role of the gut microbiome and its “meta-genome” (mG), this suggests that parasitism involves a G × G × mG interaction. Furthermore, if the gut microbiome is important to host/parasite outcomes, then the parasite must adapt to the demands of a dynamic molecular environment, e.g., the microbiome itself varies due to ontogenic development as well as physiological stresses (Koch and Schmid-Hempel, 2011; Benesh and Hafer, 2012). The gut microbiome- and/or context-dependent effects on the molecular cross-talk of host by parasite interactions could be described by the reaction norms (an inherited concept of genetics and basically applied to phenotypes, Woltereck, 1909), here it can be depicted, at least in part, as the variety of molecular patterns produced by a single G x G interaction across different gut microbiomes and/or contexts. This represents a higher complexity level compared to the G x G interactions that usually include two genetic changes in a single context or environment. Although the occurrence of such context or gut microbiome-dependent fluctuations is now assumed, their impact in altering the magnitude and the direction of the interaction has received little attention. Deeper knowledge of these complex interactions could provide a wealth of information for deciphering variability of the dynamics between host and parasite (Figure 1). Omics methodology provides an approach for efficiently detecting specific host or parasite molecular plasticity correlating with fluctuations in the gut microbiome. These methodologies also provide a gate to trace specific genes displaying broad adaptive value. Although this approach is limited to simple model systems because of its complexity, these methods could provide interesting clues to co-evolutionary processes. Moreover, the deciphering of these interactions will generate new hypotheses for the parasitic manipulation theory. The integration of the gut microbiome as a player involved in the process of the alteration of host behavior (Poulin, 2010) may even prove necessary for understanding host/parasite interactions.

### Conflict of interest statement

The authors declare that the research was conducted in the absence of any commercial or financial relationships that could be construed as a potential conflict of interest.
